# Supplementary material for: Durability and cross-reactivity of immune responses induced by a plant-based virus-like particle vaccine for COVID-19
Source: Nat Commun. 2022 Nov 12;13:6905. doi: 10.1038/s41467-022-34728-1 (PMC9653456; doi:10.1038/s41467-022-34728-1)
Supplement: Supplementary file 1 — Supplementary Information [file 41467_2022_34728_MOESM1_ESM.pdf]

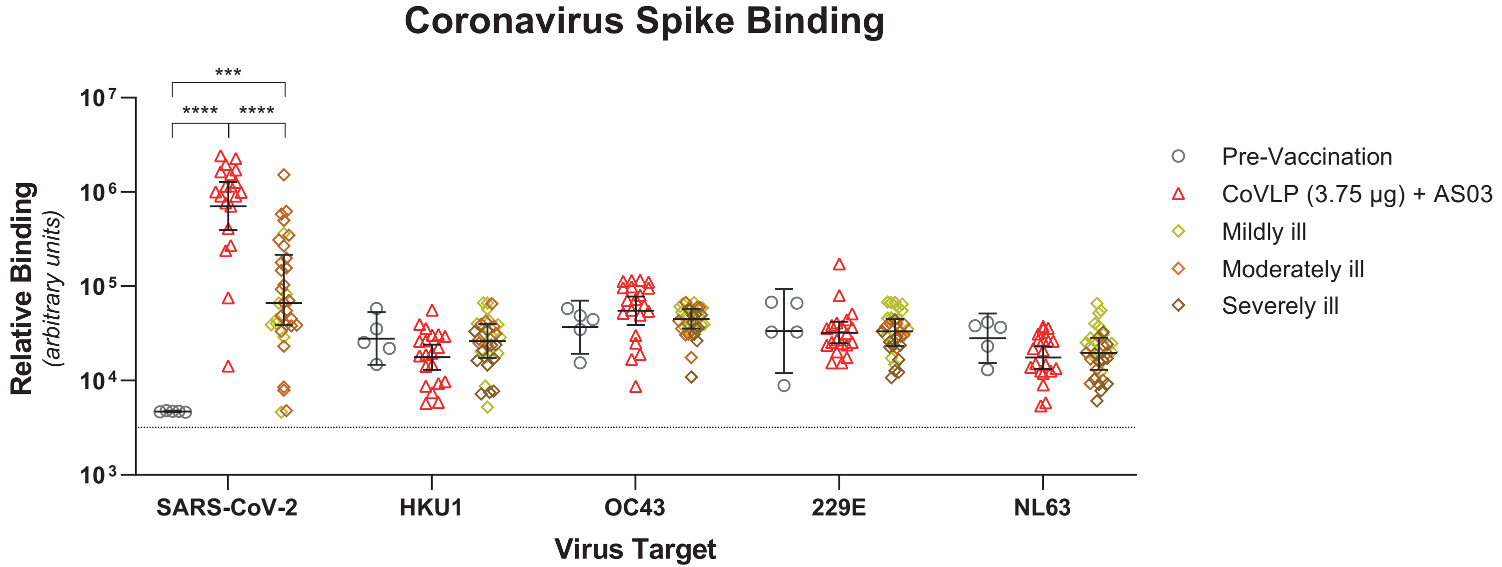

Supplemental Figure 1: Neutralizing antibody cross-reactivity to common cold coronaviruses. Binding of serum antibodies from pre-vaccinated subjects (n=5) and from D42 of subjects vaccinated (n=20) with 3.75 µg CoVLP adjuvanted with AS03 to protein S to the four common cold coronaviruses (geometric mean and 95% CI) were quantified using the VaxArray platform from InDevR, Inc. Convalescent sera or plasma collected at least 14 days after a positive diagnosis of COVID-19 (RT-pCR) from individuals whose illness was classified as mild, moderate, or severe/critical (n=35) were analyzed concurrently. Dotted line indicates mean background control values. Significant differences between sera are indicated by asterisks (***$p<0.001$; ****$p<0.0001$; One-way analysis of variance on log-transformed data. GraphPad Prism, v9.0).
