# Supplementary material for: Durability and cross-reactivity of immune responses induced by a plant-based virus-like particle vaccine for COVID-19
Source: Nat Commun. 2022 Nov 12;13:6905. doi: 10.1038/s41467-022-34728-1 (PMC9653456; doi:10.1038/s41467-022-34728-1)
Supplement: Supplementary file 2 — Reporting Summary [file 41467_2022_34728_MOESM2_ESM.pdf]

nature portfolio

Corresponding author(s): Brian J Ward

Last updated by author(s): Oct 26, 2022

Reporting Summary

Nature Portfolio wishes to improve the reproducibility of the work that we publish. This form provides structure for consistency and transparency in reporting. For further information on Nature Portfolio policies, see our Editorial Policies and the Editorial Policy Checklist.

Statistics

For all statistical analyses, confirm that the following items are present in the figure legend, table legend, main text, or Methods section.

n/a	Confirmed	
☐	☒	The exact sample size (*n*) for each experimental group/condition, given as a discrete number and unit of measurement
☐	☒	A statement on whether measurements were taken from distinct samples or whether the same sample was measured repeatedly
☐	☒	The statistical test(s) used AND whether they are one- or two-sided<br>*Only common tests should be described solely by name; describe more complex techniques in the Methods section.*
☐	☒	A description of all covariates tested
☐	☒	A description of any assumptions or corrections, such as tests of normality and adjustment for multiple comparisons
☐	☒	A full description of the statistical parameters including central tendency (e.g. means) or other basic estimates (e.g. regression coefficient) AND variation (e.g. standard deviation) or associated estimates of uncertainty (e.g. confidence intervals)
☐	☒	For null hypothesis testing, the test statistic (e.g. *F*, *t*, *r*) with confidence intervals, effect sizes, degrees of freedom and *P* value noted<br>*Give P values as exact values whenever suitable.*
☒	☐	For Bayesian analysis, information on the choice of priors and Markov chain Monte Carlo settings
☒	☐	For hierarchical and complex designs, identification of the appropriate level for tests and full reporting of outcomes
☒	☐	Estimates of effect sizes (e.g. Cohen's *d*, Pearson's *r*), indicating how they were calculated

Our web collection on statistics for biologists contains articles on many of the points above.

Software and code

Policy information about availability of computer code

Data collection	Device-associated softwares provided by Manufacturers were used for IgG (Varioskan Flash microplate reader, Thermo Fisher Scientific), pseudovirus neutralization assay (i3× plate reader, Molecular Devices) and ELISpot (ImmunoSpot S6 Universal Analyzer, Cellular Technology Limited)
Data analysis	GraphPad Prism version 9

For manuscripts utilizing custom algorithms or software that are central to the research but not yet described in published literature, software must be made available to editors and reviewers. We strongly encourage code deposition in a community repository (e.g. GitHub). See the Nature Portfolio guidelines for submitting code & software for further information.

Data

Policy information about availability of data

All manuscripts must include a data availability statement. This statement should provide the following information, where applicable:

- Accession codes, unique identifiers, or web links for publicly available datasets
- A description of any restrictions on data availability
- For clinical datasets or third party data, please ensure that the statement adheres to our policy

Medicago Inc. is committed to providing access to anonymized data collected during the trial that underlie the results reported in this article, at the end of the clinical trial, which is currently scheduled to be 1 year after the last participant is enrolled, unless granted an extension. Medicago Inc. will collaborate with its partners (GSK, Wavre, Belgium) on such requests before disclosure. Proposals should be directed to wardb@medicago.com or daoustma@medicago.com. To gain

access, data requestors will need to sign a data access agreement and access will be granted for non-commercial research purposes only.

Human research participants

Policy information about studies involving human research participants and Sex and Gender in Research.

Reporting on sex and gender	The detailed Protocol and participant demographics have been previously published and reference included in the manuscript (Ward, B.J., Gobeil, P., Séguin, A. et al. Phase 1 randomized trial of a plant-derived virus-like particle vaccine for COVID-19. Nat Med 27, 1071–1078 (2021). https://doi.org/10.1038/s41591-021-01370-1.
Population characteristics	The detailed Protocol and participant demographics have been previously published and reference included in the manuscript (Ward, B.J., Gobeil, P., Séguin, A. et al. Phase 1 randomized trial of a plant-derived virus-like particle vaccine for COVID-19. Nat Med 27, 1071–1078 (2021). https://doi.org/10.1038/s41591-021-01370-1).
Recruitment	The detailed Protocol and participant demographics have been previously published and reference included in the manuscript (Ward, B.J., Gobeil, P., Séguin, A. et al. Phase 1 randomized trial of a plant-derived virus-like particle vaccine for COVID-19. Nat Med 27, 1071–1078 (2021). https://doi.org/10.1038/s41591-021-01370-1.
Ethics oversight	Ethical approval was provided by the Advarra Institutional Review Board as well as the Health Products and Food Branch of Health Canada and the study was carried out in accordance with the Declaration of Helsinki and the principles of Good Clinical Practices. Participants were recruited from existing databases of volunteers, and written informed consent was obtained from all study participants before any study procedure. Participants were offered modest compensation for their participation in this study (time off work and displacement costs)

Note that full information on the approval of the study protocol must also be provided in the manuscript.

Field-specific reporting

Please select the one below that is the best fit for your research. If you are not sure, read the appropriate sections before making your selection.

[x] Life sciences [ ] Behavioural & social sciences [ ] Ecological, evolutionary & environmental sciences

For a reference copy of the document with all sections, see nature.com/documents/nr-reporting-summary-flat.pdf

Life sciences study design

All studies must disclose on these points even when the disclosure is negative.

Sample size	The sample size of 20 subjects in each treatment group was determine for the phase 1 study to perform the initial evaluation of the vaccine immunogenicity and detect gross differences in rates of adverse events. Given the relatively small group size and the number of experimental questions being addressed (that is, optimal dose, need for an adjuvant and best adjuvant), no formal power calculations were performed. The sample size is not large enough to detect all types, including less frequent or rare, adverse reactions. The objective of the phase 1 study was to quantify the type, percentage, intensity, duration, and relationship of short-term post-vaccination events to determine if they differ clinically among the treatment groups.
Data exclusions	Data was only excluded from the immunogenicity analysis if the subject failed to receive their second (day 21) vaccine dose resulting in a incomplete treatment. This criteria was pre-established.
Replication	The manuscript reports data generated during the Phase 1 clinical trial using validated assays.<br>The results reported in this study will be confirmed during a Phase 2/3 clinical trial (NCT04636697) which include durability and cross-reactivity assessment.
Randomization	The participants were randomized into nine groups in a ratio of 1:1:1:1:1:1:1:1:1 using a permuted block randomization schedule (pre-specified 20 participants per group).
Blinding	The subjects, the Investigators, and all personnel involved in the clinical conduct of the study (except the staff involved in the preparation and administration of the study vaccine, the quality assurance auditor, and quality control reviewers), Medicago clinical staff and medical staff involved in safety evaluations (e.g. causality assessments), and all personnel involved in sample analysis at the central and testing (Nab, ELISA, ELISpot, and flow cytometry) laboratories were blind and have no access to treatment allocation (i.e. randomization codes) for the entire duration of the study.

Reporting for specific materials, systems and methods

We require information from authors about some types of materials, experimental systems and methods used in many studies. Here, indicate whether each material, system or method listed is relevant to your study. If you are not sure if a list item applies to your research, read the appropriate section before selecting a response.

Materials & experimental systems

n/a	Involved in the study
	☒ Antibodies
	☒ Eukaryotic cell lines
☒	☐ Palaeontology and archaeology
☒	☐ Animals and other organisms
	☒ Clinical data
☒	☐ Dual use research of concern

Methods

n/a	Involved in the study
☒	☐ ChIP-seq
☒	☐ Flow cytometry
☒	☐ MRI-based neuroimaging

Antibodies

Antibodies used	Antibody used are detailed in the Method section.<br>Cellular Technology Limited (CTL) antibodies used in the ELISpot assays included: human IFNg cat # hIFNgp-2M/10, lot #I0708JMM, and IL-4 cat#hIL4p-2M/10, Lot#40721JEW
Validation	CTL antibodies are validated specifically for use in the kits they are provided with.

Eukaryotic cell lines

Policy information about cell lines and Sex and Gender in Research

Cell line source(s)	Nexelis uses ACE-2 expressing VERO cells in their pseudovirion assay.
Authentication	The cell line was authenticated by the consistent expression of ACE-2.
Mycoplasma contamination	The cell line was tested and confirmed mycoplasma free.
Commonly misidentified lines<br>(See ICLAC register)	N/A<br>Cells express ACE-2 and expression is validated so they are unlikely to be mixed with parental VERO cells.

Clinical data

Policy information about clinical studies

All manuscripts should comply with the ICMJEguidelines for publication of clinical research and a completedCONSORT checklist must be included with all submissions.

Clinical trial registration	NCT04450004 (clinicalTrials.gov)
Study protocol	The study protocol is available as supplementary material in Ward, B.J., Gobeil, P., Séguin, A. et al. Phase 1 randomized trial of a plant-derived virus-like particle vaccine for COVID-19. Nat Med 27, 1071–1078 (2021). https://doi.org/10.1038/s41591-021-01370-1.
Data collection	Detailed in the study protocol available as supplementary material in Ward, B.J., Gobeil, P., Séguin, A. et al. Phase 1 randomized trial of a plant-derived virus-like particle vaccine for COVID-19. Nat Med 27, 1071–1078 (2021). https://doi.org/10.1038/s41591-021-01370-1.
Outcomes	Detailed in the study protocol available as supplementary material in Ward, B.J., Gobeil, P., Séguin, A. et al. Phase 1 randomized trial of a plant-derived virus-like particle vaccine for COVID-19. Nat Med 27, 1071–1078 (2021). https://doi.org/10.1038/s41591-021-01370-1.
